# The assessment of the accuracy of clinical preoperative lymph node

**DOI:** 10.1097/MD.0000000000013778

**Published:** 2019-01-25

**Authors:** Yongming Qiao, Ying Wang, Peng Kang, Rui Li, Yiming Liu, Wei He

**Affiliations:** a Department of Stomatology, The First Affiliated Hospital of Zhengzhou University; b Department of Oral and Maxillofacial Surgery, Zhengzhou Stomatologic Hospital, China.

**Keywords:** elective neck dissection, head and neck cancer, preoperative assessment

## Abstract

**Purpose:**

The aim of the present study is to assess the accuracy of clinical preoperative lymph node and to define the degree of relation between the clinical preoperative assessment and histopathological examination in patients with head and neck cancer and cN+.

**Methods:**

This retrospective study was performed on 125 patients (85 males and 40 females) at the Department of Oral and Maxillofacial Surgery, the First Affiliated Hospital of Zhengzhou University, between December 2012 and December 2014.

**Result:**

Of all the patients who underwent neck dissection, 37 were found with no neck metastasis. Positive or suspected lymph nodes were detected at computed tomography (CT) in 44 and detected at ultrasonogram diagnosis (USG) in 38 of 125 patients, and the necks were assessed as normal in 55 (44%) by both USG and CT.

**Conclusion:**

Further investigation is needed to evaluate the rates of overall survival and disease-free survival of these N0 patients with neck dissection.

## 1 Introduction

Should all patients with positive pre-operative diagnostic results of neck node be given elective neck dissection? Lymph node status is one of the most important predictors of poor prognosis in head and neck cancers. Assessment and appropriate management of the clinically node-negative neck (cN0) have been a controversial issue in head and neck malignancy.^[1,2]^ However, management in patients presenting with lymph node metastases should consist of a neck dissection in which selected or all lymph node levels in the neck are removed.^[1]^ In the development of treatment paradigms, it is important to be aware that for the patients with no neck node metastases, over-treating the neck should be avoided. Therefore, accurate assessment of the lymph node status is important for the choice of treatment. Cervical lymph node metastasis staged by palpation has been demonstrated to be inaccurate.^[3]^ With the development of modern imaging modalities, the American Joint Committee on Cancer has stated that clinical staging should include physical examination as well as the results of other imaging modalities.^[4]^ Currently, ultrasound, computed tomography (CT), magnetic resonance imaging (MRI), and positron emission tomography (PET) are usually used for pre-operative assessment of the primary tumor and cervical status. These imaging techniques are comparable to each other in detecting cervical metastasis and may detect some occult nodal metastases missed by physical examination.^[5,6]^ At present, neck dissection with histopathologic examination is the most reliable staging procedure, providing important prognostic information. For these positive node patients, neck dissection is necessary in order to prevent nodal metastases and extracapsular spread.^[4]^ To date, the diagnosis of node metastases has been based mainly on size criteria; however, non-enlarged nodes may harbor malignancy, whereas benign reactive nodes may be enlarged.^[7]^

The treatment of patients with early stage, clinically node-negative oral squamous-cell cancer has been a contentious issue spanning 5 decades. For early disease, clinicians are reluctant to perform a neck dissection, because up to 85% of patients will not benefit, yet adopting a wait-and-see policy to all necks will result in a high proportion of patients subsequently developing late-stage regional failure.^[8]^ A recent study showed that the watchful waiting patient with negative node suffered a lower rate of overall and disease-free survival.^[4]^ Many studies have paid a lot of attention to clinical negative node.^[1,9]^ However, few focused on these patients with clinically positive or suspected neck lymph nodes (cN+). According to our department's surgical procedure, all the patients with malignant tumors undergo oral excision of the primary tumor with adequate margins and neck dissection. The aim of the present study is to assess the accuracy of clinical preoperative lymph node and to define the degree of relation between the clinical preoperative assessment and histopathological examination in patients with head and neck cancer and cN+.

## 2 Methods and materials

This retrospective study was performed on 125 patients (85 males and 40 females) at the Department of Oral and Maxillofacial Surgery, the First Affiliated Hospital of Zhengzhou University, between December 2012 and December 2014 (Table 1). Data were available on a consecutive series of patients with histopathologically proven squamous cell carcinoma of the oral and maxillofacial region. Exclusion criteria: patients diagnosed with any form of head and neck carcinoma in the previous 5 years; patients with other severe medical co-morbidities, known distant metastasis; patients receiving any other treatment (such as radiotherapy or chemotherapy); recurrent tumors.

**Table 1:**
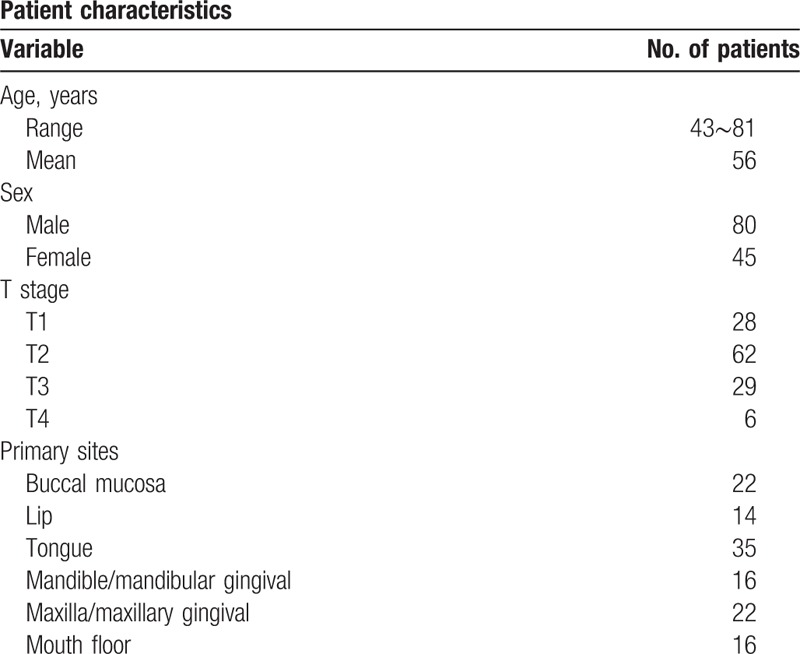
Patients’ characteristics.

Preoperative evaluation was accomplished within 2 weeks before surgery. All patients were studied using ultrasonogram diagnosis (USG) and CT of the neck. After surgery, the neck dissection specimen underwent hematoxylin and eosin staining (and immunohistochemistry staining, if necessary), node count with standard sectioning and measuring the size of the metastatic deposits in all the affected nodes.

USG was performed on all of the patients preoperatively by a set of radiologists with varying experience. Criteria for malignancy for USG investigation were as follows: size greater than 10 mm, roundness index (if length/width ratio <2:1), heterogeneous inner structure, and contour irregularity, which means extranodular involvement. CT examination of the head and neck was also performed after intravenous administration of iodinated contrast material (Omnipaque 300 [iohexol]; 9800 scanner (GE Healthcare), dose 120cc, injection rate 03 mL/s by pressure injector and scan delay:18 s). Scanning range was individually adapted. The axial images were obtained parallel to the body of the mandible from the skull base to the supraclavicular fossa with a 5-mm-thick contiguous section. Coronal reconstructions were done with 3-mm-thick contiguous slices in both soft tissue and bone windows. The criteria of metastasis for CT scanning were as follows: heterogeneous density in the node, presence of central necrosis and conglomerate lymph nodes, irregularity of the border that was accepted as extracapsular invasion, and presence of contrast material surrounding lymph node. Criteria for malignancy for CT according to the size vary according to the location of the node in different studies. At the base of previous data, we consider the size criterion to vary between 10 and 15 mm according to the other criteria.^[10,11]^

All patients were treated by neck dissections with appropriate primary tumor resections. Preoperative CT and USG findings were compared with postoperative histopathologic findings, which were accepted as the reference. The results were evaluated statistically, and the sensitivity, specificity, predictive values, and accuracy of preoperative methods were estimated.

Sensitivity was computed as the number of true positive lymph nodes/(number of true-positive + false-negative lymph nodes) × 100%. Specificity was computed as the number of true-negative lymph nodes/(number of true-negative + false-positive lymph nodes) × 100%. Positive predictive value was computed as the number of true-positive lymph nodes / (number of true-positive + false-positive lymph nodes) × 100%. Negative predictive value was computed as the number of true-negative lymph nodes/(number of true-negative + false-negative lymph nodes) ×100%. Accuracy was computed as (true-positive + true-negative)/(true-positive + true-negative + false-negative + false-positive) × 100%.

The protocol of the study was reviewed by the Institutional Review Board (IRB) of the First Affiliated Hospital of Zhengzhou University, the largest medical center of China. And all participants signed an informed consent agreement. All analyzes were performed with SPSS 21.0 software (Inc., Chicago, IL). *P* value <.05 was considered to be statistically significant.

## 3 Results

Patients’ characteristics are as per Table 1. Of all the patients who underwent neck dissection, 37 were found with no neck metastasis. Positive or suspected lymph nodes were detected at CT in 44 and detected at USG in 38 of 125 patients, and the necks were assessed as normal in 55 (44%) by both USG and CT. During these CT—suspected necks, 33 were verified with metastasis by postoperative histopathologic examination. However, only 24 of USG-detected necks were confirmed to contain malignant cells. CT: sensitivity = 33%, specificity = 68.6%, and accuracy of palpation = 45.6%; USG: sensitivity = 30.8%, specificity = 70.2%, and accuracy of palpation = 45.6%. In Table 2, the results of all methods and histopathologic investigations were presented. For these clinically negative node patients, 1 was found to have neck metastasis. There was no significant difference between CT and USG in the detection of neck node metastases. The difference between histopathological findings and USG and CT is significant. There is no relationship between T stage and node metastasis (data not shown).

**Table 2:**
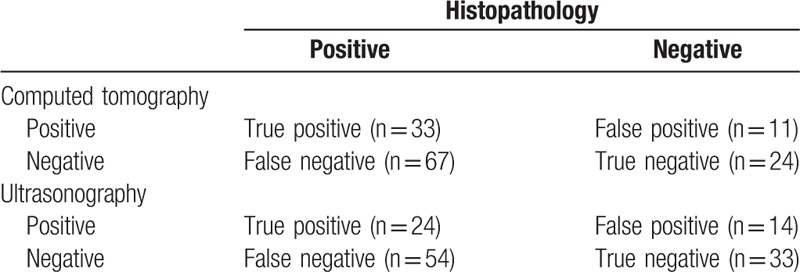
The results of all methods and histopathologic investigations.

## 4 Discussion

The presence of metastatic cervical lymph nodes is very important with regard to patient prognosis and treatment planning. The question whether imaging alone is accurate enough to guide treatment decisions in patients with head and neck malignancies depends on the ability of imaging techniques to rule out the presence of occult metastases.^[5]^ Imaging techniques such as USG, MRI, and CT, which monitor tumors or lymph node metastases by size and structural changes, have improved the staging of the neck as compared to palpation. In our research, the sensitivity of CT imaging was only 33%, at the same time the sensitivity of USG was only 30.8%. It was to say that most of the positive lymph nodes cannot be detected by preoperative CT or USG. However, the overall error rate of assessing the presence or absence of cervical lymph node metastasis by palpation has been reported as 20% to 28%, while for CT figures range from 7.5% to 28% and for MRI 16% is reported.^[12]^ Our results showed that in patients without detectable lymph nodes in the neck, there is always a risk of subclinical metastases. So our traditional procedure is that the cervical lymph nodes should be regarded as metastatic in patients with primary carcinoma of the head and neck and a neck dissection should be performed.

Cervical lymph node metastasis may be subdivided into 2 categories: overt nodal disease (clinical metastasis) and non-overt nodal disease (occult or subclinical metastasis). There are 2 classes of occult metastasis: The first consists of occult metastasis identified by traditional methods in which metastatic deposits cannot be detected on clinical or radiological examination using the most sensitive and technologically advanced procedures, but that is detected by light microscopy. A second class of occult metastasis may be designated “subpathological” or “submicroscopic” but may be detected by means of immunohistochemistry and/or molecular analysis.^[13,14]^ The critical determinant of the utility of an imaging modality for oral cavity squamous cell carcinoma is its ability to detect the presence or absence of metastatic neck disease. It is reported that palpably and radiologically negative necks (i.e. staged by USG and/or CT, and/or MRI) show occult metastatic spread in 20% to 40%, which is discovered at postoperative histopathologic examination after neck dissection,^[15]^ which was similar with our results. It is still controversial about the best treatment of the clinically negative (N0) neck. Treatment strategies include watchful waiting and treating the neck when clinical metastases develop; prophylactic irradiation; and elective neck dissection. A recent study indicated that patients with early-stage oral squamous-cell cancer, elective neck dissection resulted in higher rates of overall and disease-free survival than did therapeutic neck dissection (watchful waiting followed by neck dissection for nodal relapse).^[4]^ During this published study, the authors used USG and palpation for detecting node stage, whose examination method has been proven to have a low accuracy.^[5]^ Nodal levels with a high risk of harboring occult metastasis vary according to the site of primary tumor. The lymph nodes in these regions should be electively removed whenever appropriate as the morbidity is much less than that associated with radical neck dissection and the efficacy is comparable.

In our study, about 30% patients of N+ were found to be with no cervical metastasis, which may indicate an excessive or unnecessary treatment for these patients. In other words, the USG and CT are not the most accurate imaging modalities to detect cervical lymph node metastases. Although nodal size is considered to be the main criterion for diagnosing nodal metastasis by imaging methods, size criteria are always somewhat arbitrary for a number of reasons. First, large nodes can be reactive and not metastatic. Second, metastatic nodes are not always the largest nodes. Third, small lymph nodes can contain metastasis. To avoid the unnecessary treatment of histopathologically negative necks, a staging technique must be sensitive enough to reduce the risk of occult metastases to less than 20%, which means a negative predictive value (NPV) of more than 80%.^[5]^ The NPV of USG and CT in our study was 38% and 30%, respectively. Nowadays, more imaging techniques have been employed for assessment of node metastasis, such as USG guided fine needle aspiration cytology, diffusion-weighted MRI, 18Ffluorodeoxyglucose positron emission tomography (18FDG-PET) and so on.^[7,16]^ These imaging modalities have good diagnostic performance and higher per-neck-level sensitivity for the detection of regional nodal metastasis, compared with conventional imaging. However, due to high cost of these imaging methods, their applications were restricted in China.

It is found that the correlation of T stage with the N-stage is common to all sites, that is, the more advanced the primary tumor, the higher the percentage of patients with cervical metastasis.^[17]^ These results are substantially confirmed by our study. In patients with advanced tumors (T3–T4), who have a fairly high probability of cervical lymph-node metastases and often need neck surgery to access the primary tumors or to reconstruct the surgical defect, most head and neck surgeons will opt for elective neck dissection anyway. Yet adopting a wait-and-see policy to all necks will result in a high proportion of patients subsequently developing late-stage regional failure. Further investigation is needed to evaluate the rates of overall survival and disease free survival of these N0 patients with neck dissection. The study also has some limitations. It's just our hospital's data, not all hospitals and there is no uniform standard for accuracy or sensitivity. What's more, further investigation is needed to evaluate the rates of overall survival and disease-free survival of these N0 patients with neck dissection.

## Author contributions

**Conceptualization:** Yongming Qiao.

**Data curation:** Peng Kang, Yiming Liu.

**Software:** Wei He.

**Writing – original draft:** Rui Li.

**Writing – review & editing:** Ying Wang.
